# Indication of Mental Health from Fingertip Pulse Waves and Its Application

**DOI:** 10.1155/2018/7696458

**Published:** 2018-10-17

**Authors:** Mayumi Oyama-Higa, Fumitake Ou

**Affiliations:** ^1^Chaos Technology Research Laboratory, 5-26-5 Seta, Otsu-shi, Shiga 520-2134, Japan; ^2^PricewaterhouseCoopers Aarata LLC, 1-1-1 Otemachi, Chiyoda-ku, Tokyo 100-0004, Japan

## Abstract

This article is a comprehensive review of recent studies of the authors on the indication of mental health from biological information contained in pulse waves. A series of studies discovered that the largest Lyapunov exponent (LLE) of the attractor, which is constructed for the time series data from pulse waves, can provide as an effective indicator of mental health. A low level of LLE indicates insufficiency of external adaptability, which is characteristic of dementia and depression sufferers. On the contrary, a continuous high level of LLE indicates excessive external adaptability, and people in this condition tend to resort to violence. With this discovery, real-time display of the LLE, combined with other physiological indexes such as the autonomic nerve balance (ANB), sample entropy, and vascular age, as a reference, can enable people to conduct self-check of mental status. To this end, software development was performed in order to enable users to conduct pulse wave measurement anywhere at any time and display the analytical results in real time during the measurement.

## 1. Introduction

Biological information possesses the nature of chaos. Until now, scientists have been applying mathematical methods to the analysis of various phenomena composed of chaos. In contrast to static ones, it is usually much more difficult to explain dynamical phenomena. Thanks to the recent development of super computers, natural phenomena, such as whether conditions, have become more and more predictable. Nevertheless, even though the precision of short-term whether forecast has been improved, it is extremely difficult to perform long-term prediction. One reason is the chaotic nature of natural phenomena.

In our initial study, we collected chaotic time series data from fingertip or earlobe pulse waves, since it is convenient and does not impose a burden on the subject. Then, we applied nonlinear methods to the analysis of the data. In particular, we constructed attractors which are characteristic of chaotic data and focused on the largest Lyapunov exponent (LLE) which is a typical nonlinear statistics quantifying the divergence and instability of the attractor trajectory. Furthermore, in order to investigate the underlying biological information that produced such LLEs, we constructed mathematical models and performed measurement from subjects under general anaesthesia. We concluded that LLE possesses information of the central nervous system [1].

Based on this result, we conducted varieties of psychological experiments and studied the relationship between mental status and the LLE. As a result, we found that the LLE is related to human mental health—mental vitality and external adaptability (the ability to adapt oneself flexibly to the external environment). In particular, we have come to realize that the LLE can provide as an effective indicator in the studies of dementia, depression, and measures to prevent work errors, all of which are related to the external adaptability [2–5].

In the following main parts of this article, we start with the chaotic nature of fingertip pulse waves, and then explain, by mathematical modelling and anaesthetic experiment, what information is indicated by the LLE that is obtained from the fingertip pulse waves. Next, we explain the calculation of the LLE, and the values of high frequency (HF), low frequency (LF), and the autonomic nerve balance (ANB), which are also used in our studies and are calculated from the heartbeat data in the same experiment. Furthermore, we discuss the effectiveness of the sample entropy computed from the entropy of vascular blood flow.

In order to study LLEs of a group of people simultaneously, we developed a system that enabled us to measure the pulse waves of more than one subject at the same time. “Lyspect” (developed by the Chaos Technology Research Laboratory, or CTRL) is such a system that can visualize the nonlinear analytical results on a PC screen with graphs and figures. We will then describe the functions of “Lyspect.”

Moreover, we will introduce “Alys” (developed by CTRL), a mobile application portable for Android tablets and smartphones, which can display both LLE and ANB in real time during the measurement [6].

## 2. Biological Information and Chaos

A living body is an extremely huge complex system that takes in information from the ever-changing internal and external environment—organs, blood flows, as well as the outside world—and operates following the instructions given by the cerebrum.

**Figure 1:**
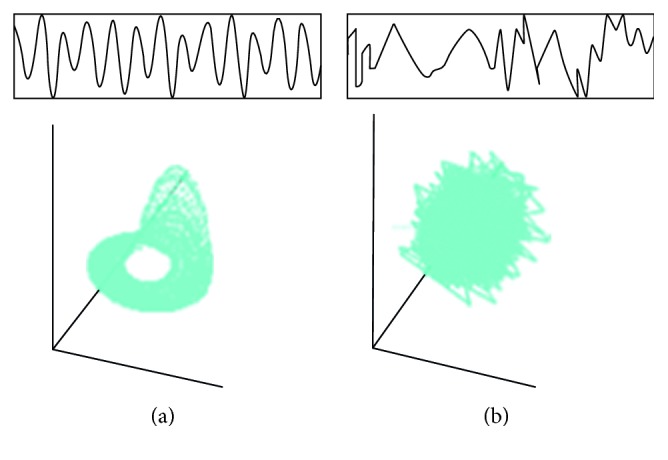
Difference between (a) chaos and (b) randomness.

On the contrary, many phenomena in the world are observed to be random. What is the difference between randomness and chaos? Figure 1 compares two attractors—one of chaotic data and the other of random data. The difference is clear. If the biological information was random, it would be unable to support life.

In contrast with a random system, a chaotic system is driven by deterministic rules instead of probabilistic factors. Even though the probabilistic factors do not exist, the deterministic chaos produces complicated behaviour due to the nonlinearity that the system itself possesses. Chaos has several characteristics, such as sensitive dependence on initial conditions, trajectory instability, long-term unpredictability, and short-term predictability. However, even though the trajectory instability occurs owing to minute disturbance, the geometric structure of the attractor, which expresses the stationary behaviour in the state space, generally remains unchanged. Moreover, regarding the sensitivity to initial conditions, deterministic prediction is possible, provided the term is short enough.

Fingertip pulse waves are proved to be deterministic chaotic time series data that human body generate. Concerning the evidence, in particular, the following 4 conditions are satisfied:

1. The data possess no characteristic frequency component, but wideband power spectra
2. Adjacent trajectories that pass through the subspace of the attractor go in the same direction
3. The LLE computed from the attractor is positive
4. Nonexistence of white noise can be confirmed from correlation dimension (fractal dimension). To this end, we can apply the Grassberger–Procaccia method (G–P method) to the time series data

**Figure 2:**
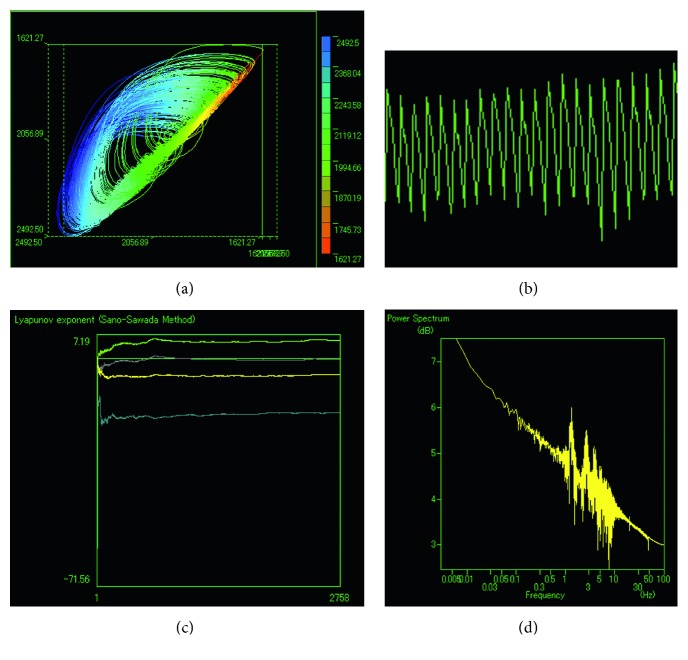
Fingertip pulse wave (b), power spectra (d), attractor (a), and 4 LLEs ((c), where the calculation results are *λ*_1_ = 5.53, *λ*_2_ = −0.46, *λ*_3_ = −5.26, and *λ*_4_ = −17.09).

As can be seen from the Figure 2, fingertip pulse waves satisfy all the above 4 conditions, and thus they are chaotic time series data.

## 3. Measurement and Analysis of Chaotic Time Series Data

**Figure 3:**
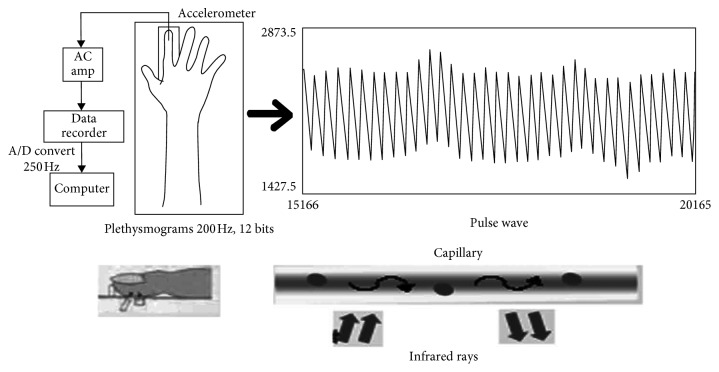
Measurement of pulse waves.

Capillaries, which are the smallest veins and arteries, are abundant in fingertips and earlobes.  Figure 3 shows the process of measuring pulse waves. We take 200 Hz analogue data from the fluctuation of haemoglobin in the red blood cells by using an infrared sensor, and then convert them to data with no less than 10 bits, by analogue-to-digital conversion.

Concerning the time period for the measurement, though a short time period can produce a result, it is appropriate to secure at least 1 minute in order to improve the precision of the autonomic nerve balance (ANB) value, which is computed from the heartbeat. Besides, considering the chaotic feature of human body, it is not reasonable to perform the measurement for only one time. Therefore, it is recommended to perform sufficient times of measurement with sufficient time period, according to the purpose [7].

In the following, we introduce the nonlinear analysis of pulse waves and the largest Lyapunov exponent.

### 3.1. Construction of Attractor

As discussed earlier, pulse wave data are chaotic time series data. The structure of dynamical systems that follow deterministic patterns can be described by several state variables. This is realized by Takens' embedding theorem.

To perform the embedding, time delay and embedding dimension are required. To obtain these, generally the autocorrelation coefficient and mutual information of the time series data can be used as a standard [8].

### 3.2. Calculation of LLE

**Figure 4:**
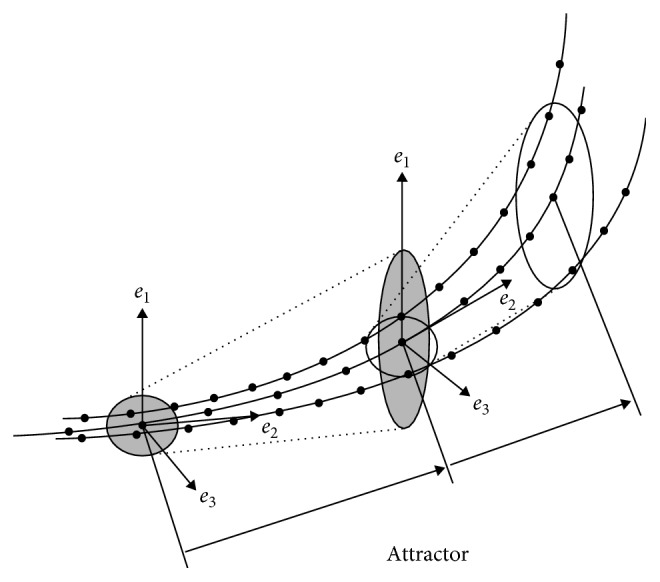
Calculation of Lyapunov vector from changes of the attractor trajectory (3-dimensional illustration).

Regarding the calculation of LLE, the indicator of trajectory instability of the chaotic time series data, the optimal choice of the embedding dimension is 4 [9, 10]. To illustrate the process of the calculation, we depict the attractor conceptually with dimension 3 (Figure 4). Given a small ball as the initial value, a mapping operation stretches out the ball in direction *e*_1_, and then presses it back in direction *e*_3_. Therefore, the ball is transformed into an ellipse. Let *λ*_1_, *λ*_2_, and *λ*_3_ denote the logarithms of the magnification ratios per unit of time in directions *e*_1_, *e*_2_, and *e*_3_, respectively. Here, *λ*_1_, *λ*_2_, and *λ*_3_ are called Lyapunov exponents, and the collection (*λ*_1_* λ*_2_* λ*_3_) is a Lyapunov vector.

In our study, we calculate the Lyapunov vector several times and take the average value. As mentioned above, the embedding dimension for the pulse waves is 4. Thus we have calculated the 4-dimensional Lyapunov vector (*λ*_1_* λ*_2_* λ*_3_* λ*_4_). Without loss of generality, let *λ*_1_ be the largest of the four components, and this is the largest Lyapunov exponent we have obtained.

The following is a more rigid explanation. Consider the time series data denoted by

(1) $$\begin{array}{c} x\left(i\right),\;i=1,2,\ldots . \end{array}$$

By using the method of delays, the phase space is reconstructed with vectors represented as

(2) $$\begin{array}{c} X\left(i\right)=\left(x\left(i\right),x\left(i-\tau \right),\ldots ,x\left(i-\left(d-1\right)\tau \right)\right)=\left\{x_{k}\left(i\right);\;\;k=1,\ldots ,d\right\}, \end{array}$$

where *τ* is a constant delay, *d* is the embedding dimension, and *x*_*k*_(*i*) is defined as

(3) $$\begin{array}{c} x_{k}\left(i\right)=x\left(i-\left(k-1\right)\tau \right),\;k=1,\ldots ,d. \end{array}$$

Regarding the fingertip pulse waves, previous studies [9, 10] have shown that the optimal choices for the constant time delay and the embedding dimension are

(4) $$\begin{array}{c} \tau =50\text{ ms,} \end{array}$$

and as mentioned above,

(5) $$\begin{array}{c} d=4. \end{array}$$

*X*(*t*) evolves with time with the initial trajectory *X*(0). Then the LLE (or denoted as *λ*_1_, as above) is given by the following formula:

(6) $$\begin{array}{c} \text{LLE}=\underset{t⟶\infty }{\lim}\underset{\varepsilon ⟶0}{\lim}\frac{1}{t}\log\frac{\left|\delta X_{\varepsilon }\left(t\right)\right|}{\left|\varepsilon \right|}, \end{array}$$

where

(7) $$\begin{array}{c} \delta X_{\varepsilon }\left(t\right)=X\left(t\right)-X_{\varepsilon }\left(t\right), \end{array}$$

represents the divergence of the trajectories, with initial condition of

(8) $$\begin{array}{c} \varepsilon =X\left(0\right)-X_{\varepsilon }\left(0\right). \end{array}$$

In our studies, the method proposed by Rosenstein et al. in 1993 [11] is applied for estimating the LLE. Moreover, the value of LLE is normalized to a range of 0–10 in the display of our measuring device.

**Figure 5:**
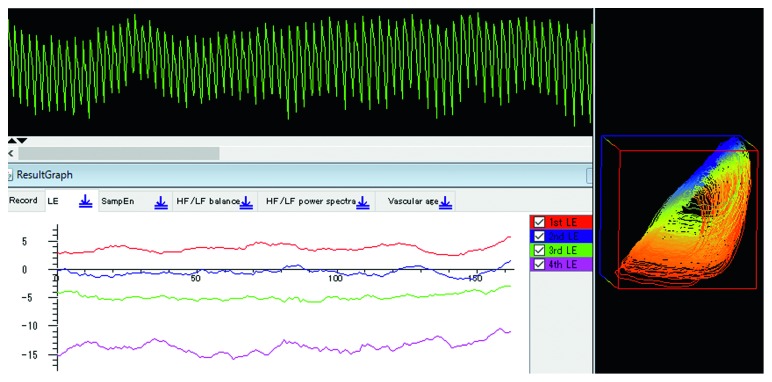
Pulse waves, attractor, Lyapunov exponents (“1st LE” to “4th LE,” in different colours, represent *λ*_1_ to *λ*_4_, respectively) including the LLE (red).

Figure 5 shows the attractor, components of the Lyapunov vector (*λ*_1_* λ*_2_*λ*_3_*λ*_4_), and the largest Lyapunov exponent (red), displayed by our software “Lyspect.”

## 4. Meaning of LLE Calculated from Pulse Waves

### 4.1. Investigation

To investigate the meaning of the LLE, we applied the following 2 methods:

  Method 1: Mathematical modelling
  Method 2: Observation of changes under general anaesthesia

**Figure 6:**
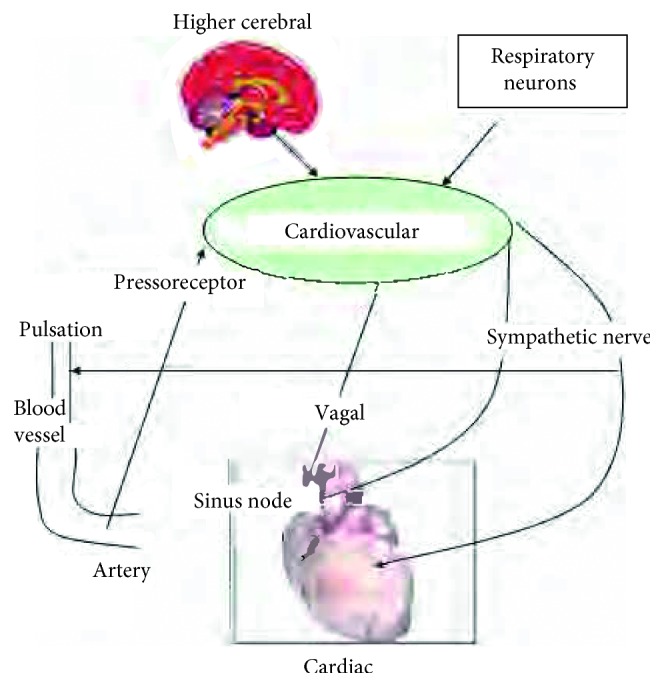
Factors considered to have an impact on the pulsation.

The mathematical modelling approach in the study of chaotic behaviour is widely applied [12]. In regard to the subject of our study, we considered the following factors [13] Figure 6.

We performed simulations of the factors considered to have an impact on the pulsation. By using the inverse Fourier transform, we combined the waves sent by each of the factors. Then, we compared the combined wave with the pulse wave obtained from the measurement.

**Figure 7:**
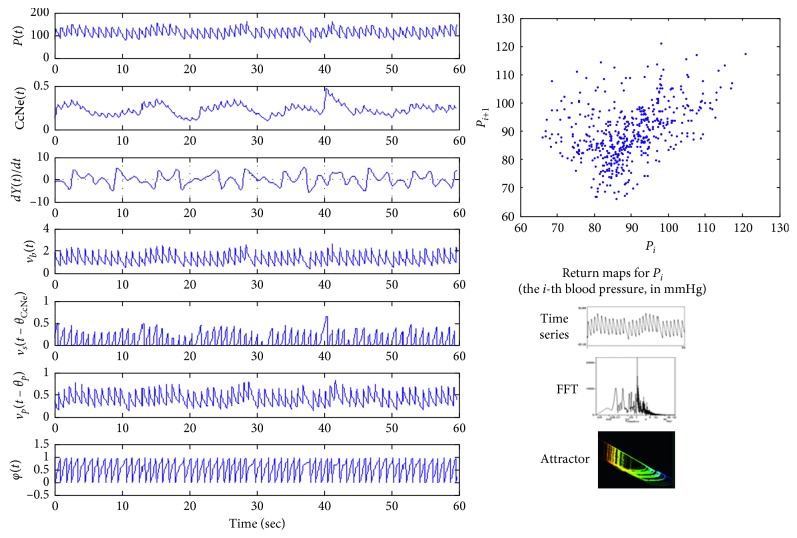
Simulation based on the model that considered the central nervous system.

In the initial model, we did not incorporate the Duffing oscillator which is related to the central nervous system. Consequently, regarding the Mayer wave and RSA wave resulted by the fast Fourier transform (FFT), we did not obtain sufficient results. Thus, we modified the model by adding a mathematical expression related to the central nervous system, as shown in Figure 7, and finally we obtained the wave close to pulse wave.

The result of the simulation based on the mathematical model showed that the pulse waves possess information of the central nervous system [13].

Next, we explain Method 2, the observation of changes in the LLE when the subject is under general anaesthesia. We have found, by mathematical models, that pulse waves contain the information of the central nervous system. To further obtain empirical data, we conducted an experiment with the subjects under the condition that their central nervous systems do not function. To this end, with the consent of a group of cancer patients from a hospital in Kyoto, we managed to collect their pulse waves which were taken from the time before they received the general anaesthesia for a surgical operation to the end of the operation.

**Figure 8:**
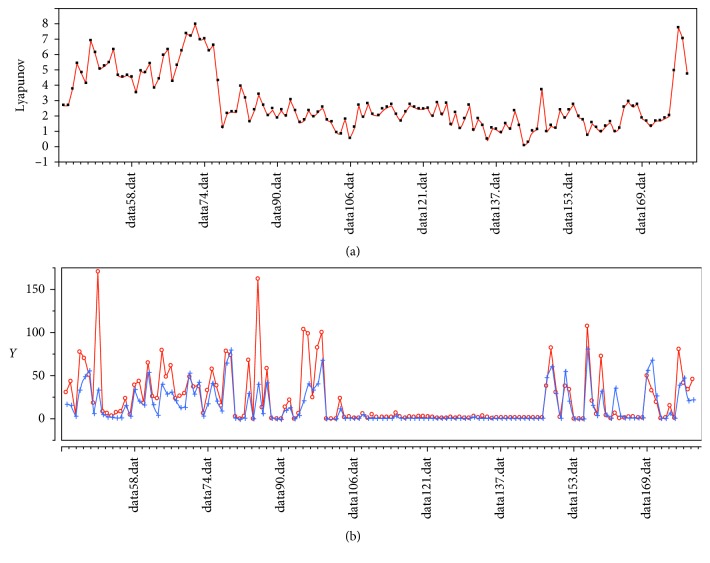
(a) Changes in values of LLE. (b) Parasympathetic nerve and parasympathetic nerve under general anaesthesia.

Changes in values of the LLE, sympathetic nerve, and parasympathetic nerve are sketched in Figure 8. These values were calculated from data output by the pulse wave-measuring device, based on a timetable of the anaesthesia provided by the doctor. We can clearly observe that the value of the LLE fell drastically when the anaesthesia was administered, and the low level continued until the anaesthesia lost effect. However, note that the value did not turn to zero. The reason is considered to be the ceaseless function of the heart.

As we have clearly seen, from both the simulation based on model and the experiment under anaesthesia, the LLE is a value related to the central nervous system [14, 15].

### 4.2. LLE as an Indicator of Mental Health

As we have already discussed in Section 2, the LLE represents changes of the attractor trajectory, which always fluctuates. The fluctuation is essential in the indication of human mental health. Specifically, we are going to show data and analytic results from varieties of experiments. The indication of mental health, simply put, is the condition that the LLE keeps fluctuating up and down within a moderate range, namely, the condition of moderate fluctuation. No fluctuation, or no change in the value of LLE, indicates indifference to the external environment and inability to adapt oneself to the external changes. Furthermore, the condition when LLE keeps in a low level (between 0 and 2, according to the normalized range as mentioned in Section 3.2) indicates that the external adaptability is insufficient, which is characteristic of many patients with mental illnesses such as dementia and depression. On the contrary, a continuous high level of LLE (between 8 and 10) indicates excessive external adaptability, and the people in this condition tend to resort to violence. Such people are recommended to be self-aware of this potential danger.

Therefore, a person can be judged mentally healthy if his or her LLE always fluctuates up and down within a moderate range. Moreover, mentally healthy people are able to adjust their LLEs upwards or downwards. In contrast, however, mentally unhealthy people may not. For example, for social withdrawals which have become an object of public concern, their LLEs are usually kept at a low level but sometimes drastically go up (from 1 to 10), which reflects their inability to control their mental status.

In order to quantify our mental health and flexibly, we can equip an Android tablet or a smartphone with a sensor for the measurement and an application for the analysis and display of the analytical results. In particular, “Alys” can display the range of LLE values in colour, which is convenient for users to check their mental status without the limitation of time and place. Mentally healthy people may control themselves by referring to the colours.

## 5. “Lyspect” for the Analysis of Chaotic Time Series Data

In this section, we give a detailed introduction of “Lyspect,” the software for analysis of chaotic time series data [6]. Installed on a PC, “Lyspect” excels in the analysis and visualization of the analytical results. Besides, the software “Lyspecting” is built in “Lyspect,” which enables users to see the displayed condition while performing the measurement. In the following, we describe the main items for calculation, analysis, and display with “Lyspect.”

### 5.1. Value of LLE

The LLEs, calculated from changes of the attractor trajectory, are displayed in time series. To display one LLE value as a representative, the average and standard deviation of the calculated LLE values can be used. As mentioned in Section 3.2, the value is normalized to a range of 0–10. Generally, the average LLE of a mentally healthy person is around 5.

### 5.2. Value of Autonomic Nerve Balance

Since pulse waves contain heartbeat information, which is related to the activity of the autonomic nervous system, we also obtain the values of high frequency (HF, 0.15–0.40 Hz) and low frequency (LF, 0.04–0.15 Hz) from the analysis of the heartbeat frequency, and display them in time series. Based on HF and LF, we introduce the value of autonomic nerve balance (ANB), defined as a normalized index ranging from 0 to 10:

(9) $$\begin{array}{c} \text{ANB}=\frac{10\;B}{3.5}, \end{array}$$

where

(10) $$\begin{array}{c} B=\frac{\ln\left(\text{LF}\right)}{\ln\left(\text{HF}\right)}. \end{array}$$

This computation method, a registered U.S. patent [16], has been embedded in the measuring device used in our studies.

The necessary time period for the measurement should be at least 1 minute, in order to perform integration in the frequency domain. When the sympathetic nerve keeps active, stress tends to build up, which influences mental health. Regarding the result of the ANB, a value less than 5 indicates predominance of parasympathetic nerve, while a value over 5 indicates sympathetic predominance.

**Figure 9:**
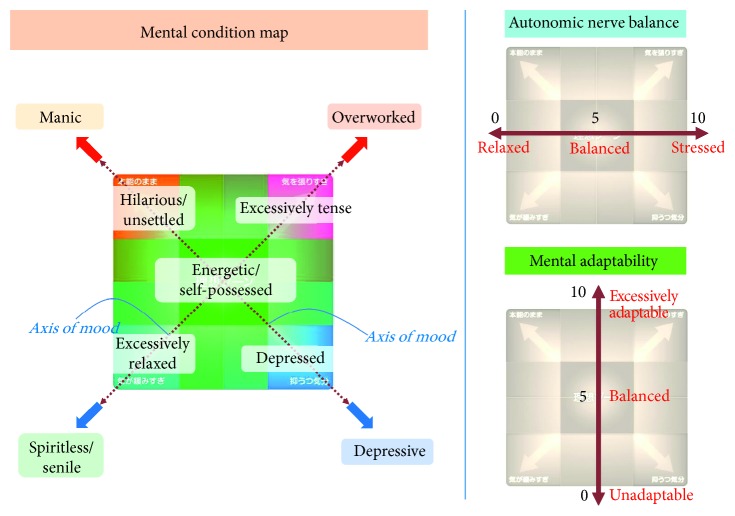
Mental condition map using LLE and ANB.

Displaying the LLE and ANB with 2 axes can make the analytical result for mental health more clear.  Figure 9 presents maps of mental condition using the values of the LLE and ANB calculated by “Lyspect.” These are taken from the display screen of “Lifescore Quick” (developed by WINFrontier Co., Ltd.) in which the LLE computational engine of “Lyspect” is incorporated. “Lifescore Quick” has already come into the market. Users can clearly observe their condition of mental health from the zones that are displayed according to the analytical results.

Since the product is currently sold in Japanese market only and the English version is not available, here is an explanation of the “mental condition map.” The upper half of the cubic map shows mental conditions when the LLE is high, namely, the normalized LLE value is over 5, while the lower half of the map corresponds to the opposite case, that is when LLE is low. Similarly, the left half of the map indicates the condition when the ANB is low, and vice versa for the right half.

Therefore, the upper left red arrow points at the case when LLE is high but ANB is low, and it has been found that people under such condition are unsettled, which is a syndrome of mania. The other three combinations can be explained similarly. When both LLE and ANB are high, as the upper right red arrow points at, nerves are at full stretch, which is a characteristic of overwork. For the lower half, when both LLE and ANB are low, as the lower left blue arrow points at, strain is relieved, which is often seen in lethargic or senile people. The lower right blue arrow points at the condition when LLE is low but ANB is high, which indicates that the individual is feeling depressed.

### 5.3. Sample Entropy

In our study, the sample entropy is computed from the entropy of vascular blood flow. Suppose there are two sequences that are similar for certain points, within a given tolerance. We can find the probability of the occurrence of this event. Further, we can compute the conditional probability that these two sequences remain similar when one consecutive point is added to each of them. As a conventional method for studying the complexity in biological time series, the sample entropy is defined as the reciprocal of the natural logarithm of this conditional probability. For the time series data *x*(*i*), *i*=1,2,…, *N*, as given by (1) in Section 3.2, the sample entropy is computed as

(11) $$\begin{array}{c} \text{SampEn}\left(m,r,N\right)=-\ln\frac{A^{m}\left(r\right)}{B^{m}\left(r\right)}. \end{array}$$

In the above expression, *B*^*m*^(*r*) denotes the probability that two sequences match for *m* points, *A*^*m*^(*r*) denotes the probability that these two sequences will match for *m*+1 points, and *r* is the given tolerance. Here, *m* is also treated as the length of subsequences of the data sequence *x*={*x*(1), *x*(2),…, *x*(*N*)} [17].

For a healthy subject, when the length *m* is changed from 2 to 10, the sample entropy tends to decrease as the tolerance *r* increases. However, illnesses can cause the sample entropy to increase. Therefore, it is possible to discover potential illnesses that cannot be indicated by the LLE and ANB, by checking the behaviour of sample entropy.

### 5.4. Vascular Age

Vascular age is generally of interest in the study of physiological and biological information. It can be computed using the second derivative of photoplethysmogram (SDPTG) [18], and the vascular age can be estimated by the average of the results obtained from such computation conducted thousands of times, provided the pulse waves of the subject are stable during the measurement. However, if unstable pulse waves are taken in, the precision of the computational results will fall.

In order to overcome this drawback, in “Lyspect,” the value adjusted with median and width is applied, as shown below.

(12) $$\begin{array}{c} \sigma =\frac{10}{1+\exp\left\{-\left(2.857N-100\right)/30\right\}}, \end{array}$$

where *N* is the physical balance of the blood vessel, computed using the SDPTG. Here, *N* is comparable to the actual age: a high value indicates unbalanced function of the vessel and vulnerability to sclerosis, while a low value indicates plasticity of the vessel. Also note that *σ* is monotonically increasing with respect to *N*, ranging approximately from 0 to 10 as *N* goes from 0 to 100.

### 5.5. Representative Values

**Figure 10:**
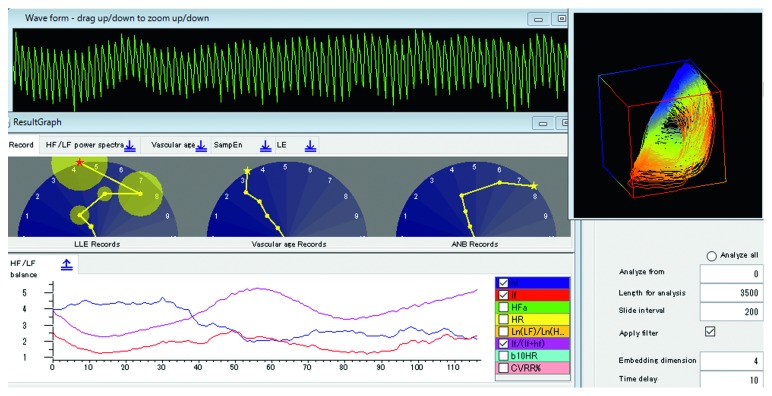
Display of analytical result (one part).

In “Lyspect”, as mentioned in Section 5.1, one LLE value is displayed as a representative. For ANB and vascular age, representative values will also be displayed.  Figure 10 is the display screen of “Lyspect.” In the middle panel, three semicircles display, from the left, LLE, vascular age, and ANB, respectively. All of them are normalized to a range of 0 to 10.

For the LLE, the standard deviation of each value is displayed with a small yellow circle. A big circle suggests a wide interval for the estimated value, whereas a small circle means the calculated values are centred on the mean.

Concerning the vascular age, the result is shown as the value that the needle is pointing at. As explained in Section 5.4, the precision is highly dependent on the stability of the pulse waves during the measurement.

Regarding the ANB, as stated in Section 5.2, when the needle is pointing at the right half of the semicircle, the value is over 5, which indicates sympathetic predominance and vice versa.

### 5.6. Other Functions

In addition to the graphs, “Lyspect” also outputs numerical results, which can be imported into Excel for analysis.

Besides, on the bottom panel, users can select the representative value they like, and when the recording is selected by right-clicking, changes of these representative values will be displayed in real time (Figure 10).

There are other functions that are convenient for researchers, such as filtering of the collected pulse waves and download of the data and graphs.

## 6. Psychological Experiments Using the LLE and Indication of Mental Health

In this section, we show several results of psychological experiments using the LLE. In each experiment, unless otherwise specified, we conducted the measurement of fingertip pulse waves with a 3-minuite time period for each time.

### 6.1. Degree of Dementia and Communication Skills of the Aged

#### 6.1.1. Subjects

179 aged people (40 males and 139 females) from 3 welfare facilities for the elderly in Shiga Prefecture, Japan.

#### 6.1.2. Experiment Time

From August to November 2003.

#### 6.1.3. Experiment Process

For each subject, 3 times of measurement were performed in a relaxed condition at room temperature (25°C). Blood pressure, pulse, and body temperature were taken before the measurement.

#### 6.1.4. Indexes

We were provided data of 5-stage assessment results showing the degree of dementia judged by the doctor. We also obtained data of the results of a 3-stage assessment concerning ADL (activities of daily living), composed of 7 items, prepared by the care managers. Then, we investigated the relationship between these data and the LLE calculated from the pulse waves [15, 19].

#### 6.1.5. Result

**Figure 11:**
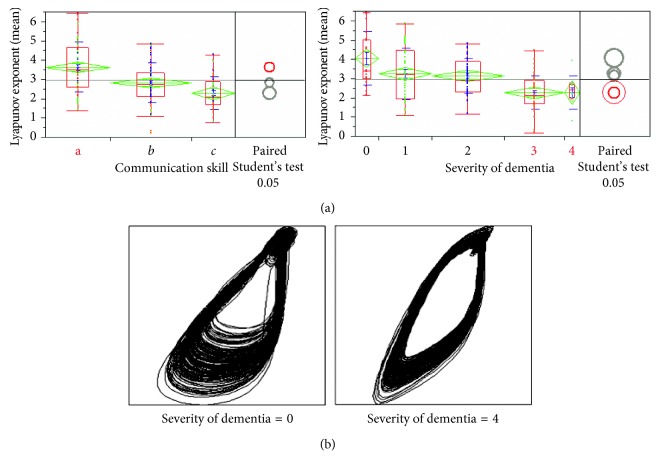
(a) LLE (vertical axis), communication skill (3-stage result), and degree of dementia (5-stage result); (b) attractors (2-dimensional projection) with degree of dementia 0 (normal) and 4 (severe).

Significant relationship was found between the LLE and the degree of dementia as well as the communication skill indicated by the ADL results (Figure 11). From Figure 11, we may also observe the difference in the degree of dementia from the attractors.

### 6.2. Children's Pulse Waves and Their Mothers' Attachment

#### 6.2.1. Subjects

Two hunded forty-two little children aged from 0 to 5, from nursery schools in Osaka and Himeji Prefectures, Japan. The details concerning the number of subjects for each age and sex are given in Table 1.

#### 6.2.2. Experiment Time

From January 2004 to March 2005.

#### 6.2.3. Experiment Process

For each subject, measurement of fingertip pulse waves was performed 2 times in a relaxed condition at room temperature (25°C), and the time period for each time was 1 minute. We did not adopt the default 3 minutes but had to shorten the measurement time, because some of the children could not help moving their hands, which may cause measurement errors.

#### 6.2.4. Indexes and Results

**Figure 12:**
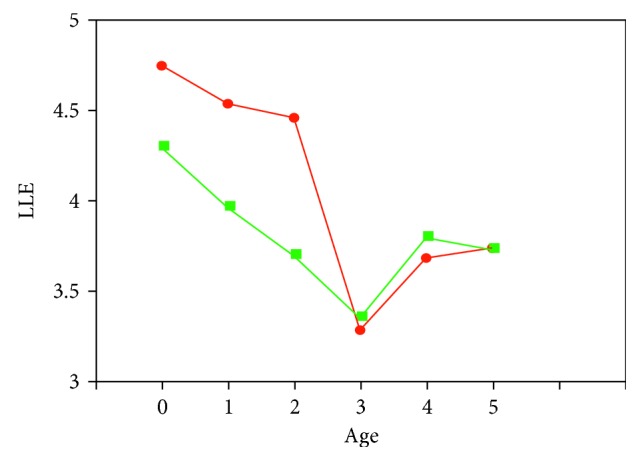
Average LLE values for each age of the children.

Compared with the other ages, the children aged 3 showed a significantly low LLE value, as illustrated in Figure 12. The Student's *t*-test showed that the relationship between the LLE value and age is statistically significant at 5% level of significance.

**Figure 13:**
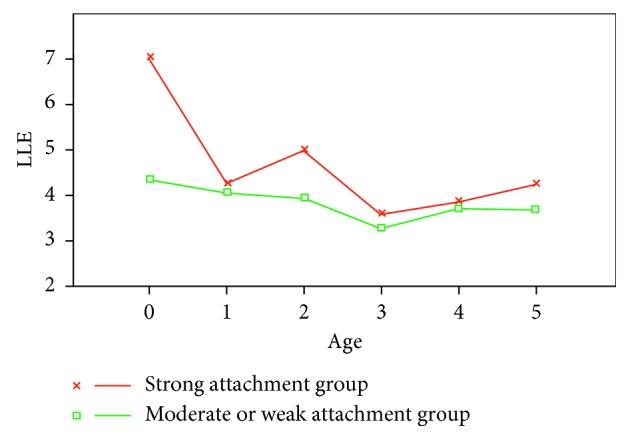
Average LLE values for each age of the children, in two groups with different degrees of attachment.

Furthermore, we investigated their mothers' attachment for the children, our subjects. We collected the results of degree of attachment through questionnaires for the mothers, where the indexes of the Maternal Attachment Inventory (MAI, cf. [20]) were applied. We separated the children into 2 groups based on the degree of their mothers' attachment—strong attachment and moderate or weak attachment. In order to observe the behaviour of LLE values in each group, we reflected the grouping to Figure 12 and then obtained Figure 13. For both groups, the Student's *t*-test resulted in a statistically significant relationship between the LLE value and age at 5% significance level [21, 22].

### 6.3. Employees' Pulse Waves and Their Degree of Fatigue

#### 6.3.1. Subjects

Twelve employees of a certain company.

#### 6.3.2. Experiment Time

Our measurement was assisted by a Hitachi company, in February, 2005. The measurement for each subject was carried out in the morning (immediately after the employee came to the office of the company), daytime (more than 1 hour after lunch), and evening (immediately before the employee left the office).

#### 6.3.3. Experiment Process

For each subject, measurement of fingertip pulse waves was performed in a relaxed condition at room temperature (25°C).

#### 6.3.4. Indexes and Results

**Figure 14:**
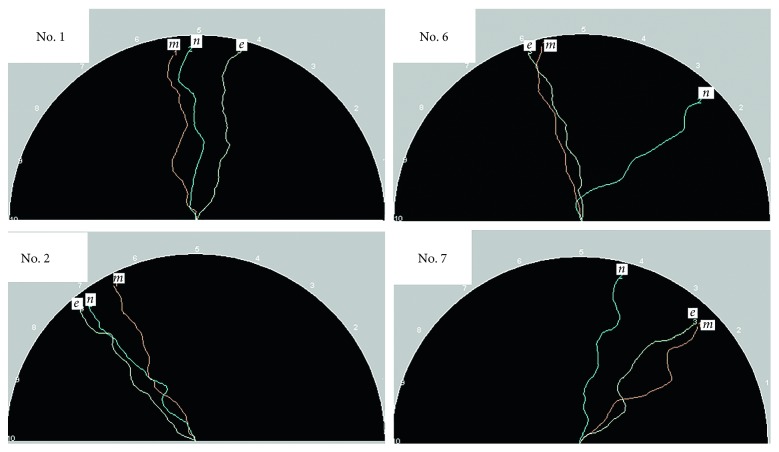
LLEs from pulse waves measured in different periods of time.

In addition to the LLEs computed from the pulse waves, we also collected the employees' fatigue indexes through questions. Then, we analysed the relationship between the fatigue indexes and the LLE values.  Figure 14 shows several patterns of the behaviour of LLEs, which were from pulse waves taken in different time periods.

We observed that those with a low LLE in the day time developed a tendency to feel depressed or uneasy. From Table 2, we can observe the significant inverse correlation between the LLE in the day time and the level of anxiety (correlation coefficient = −0.7279) and between the day-time LLE and the tendency toward depression (correlation coefficient = −0.7014). In other words, a low LLE was observed to accompany strong tendency toward depression and higher level of anxiety.

Due to the frequent occurrence of mental disorders nowadays, mental health management has been playing an increasingly important role in the workplace. As an indicator of mental health, LLE can be useful not only for the self-check of the employees but also for the employers to assist in their human recourse management [23].

### 6.4. Changes in LLE during a Calculation Task

**Figure 15:**
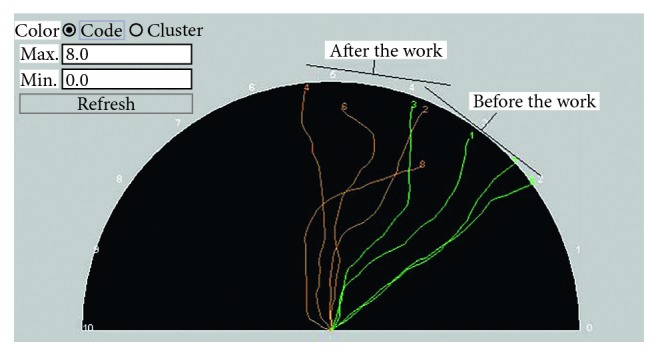
Changes in LLEs before and after the Kraepelin test.

We performed a 15-minute Kraepelin test 2 times on each of the subjects of age of twenties and of forties, and observed changes in his or her LLE before and after the test. For both age groups, we found that the LLE increases after the test. According to the subjects, they felt more clear-headed after the test.  Figure 15 shows the change in LLEs [24].

### 6.5. LLE and the Occurrence of Errors during a Monitoring Task

**Figure 16:**
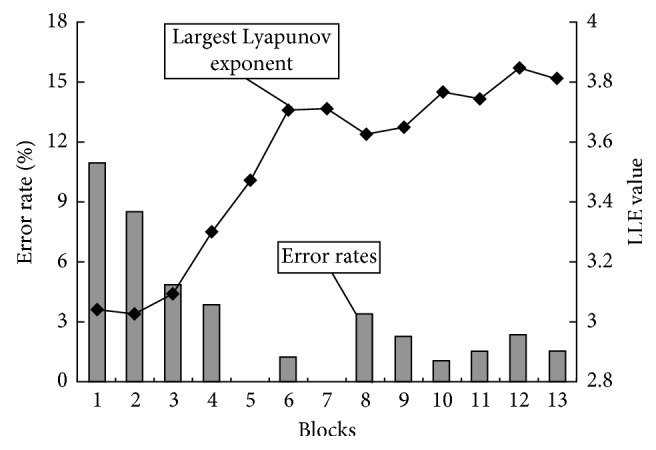
Changes in LLEs and error rates during a monitoring task.

For the purpose of experiments on human errors, we developed a device which enables subjects to perform monitoring tasks with a PC. Then we investigated the relationship between the LLE and error rates of the subjects. The subjects were asked to perform monitoring tasks with changing environments on the PC screen. For all the experiments, we observed that higher error rates corresponded to lower LLEs, as shown in Figure 16. The line chart and bar chart represent the LLEs and the error rates, respectively [25].

From the results of the psychological experiments 6.3, 6.4, and 6.5, as demonstrated above, we have found that the best time to set to work or go into action is when one's LLE is high to some extent.

### 6.6. Changes in Emotions during a Painting Work

**Figure 17:**
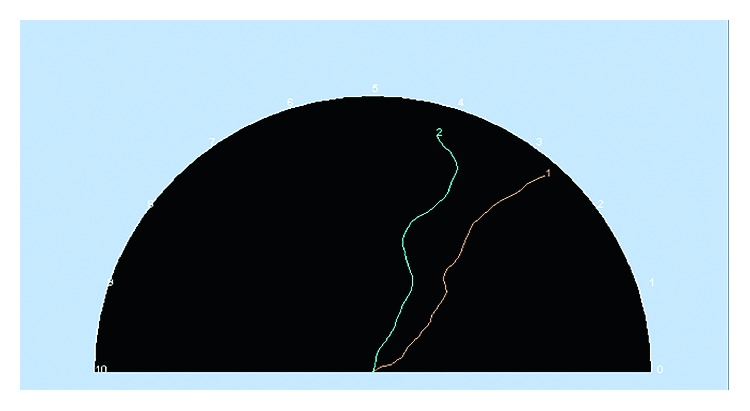
Changes in the LLE due to the painting work.

By performing similar experiments, we found that the LLE tended to increase owing to the activity of painting.  Figure 17 displays the LLEs of a painter when she was at rest (in orange) and when she started to paint after 3 minutes [26].

### 6.7. Changes in the LLE during Childbirth

**Figure 18:**
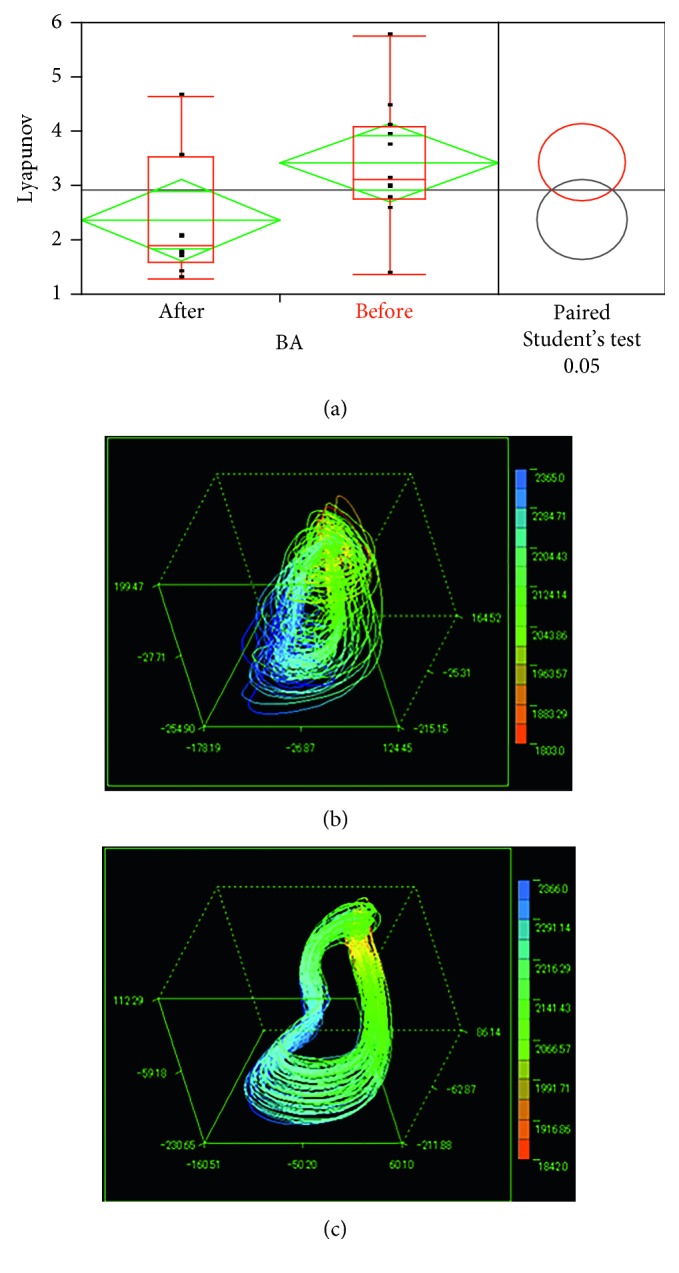
Changes in the LLE during childbirth: significant difference (a) and attractors before childbirth (b) and after childbirth (c).

In cooperation with the obstetrics and gynaecology department of a hospital in Nara Prefecture, Japan, we performed similar experiments and observed changes in the LLE of pregnant women before and after their childbirth. We compared the LLE values within 1.5 hours before the childbirth and those within 1.5 hours after the childbirth, as shown in Figure 18. The Student's *t*-test gave a result of *p* < 0.05, meaning that the higher LLE was significantly related to the before-childbirth case. This indicated that in preparation for their childbirth, pregnant women tend to strive to adapt themselves to the crucial external condition [27].

### 6.8. LLE and Laughter

**Figure 19:**
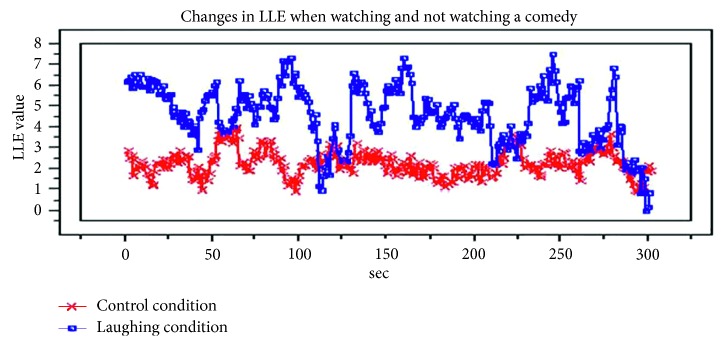
Comparison of LLE: when the subject is laughing (in blue) and not (the control, in red).

By similar means, we compared the LLEs of students watching a comic video, and those of students at rest (as a control group). The results are shown in Figure 19. As the saying goes, laughter is the best medicine. We observed that laughter caused the LLE to increase. Related work can be found on the homepage of the “Loud Laughter and Health Programme” ([28] in Japanese, http://www.v-1.co.jp/owarai/koyo.html) [27].

From the above 8 psychological experiments, we further substantiated our conclusion, reached by the result of mathematical modelling and anaesthetic experiment, that the LLE possesses information of the central nervous system.

### 6.9. Experiments on Communications at an Old Age Home

In cooperation with an old age home, we measured the pulse waves of a caregiver and several bedridden aged people when the caregiver continually spoke to each of a number of aged people about a topic of interest for 5 minutes. For each 5-minute time period, the measurement on the pair of the caregiver and an aged person was conducted simultaneously.

**Figure 20:**
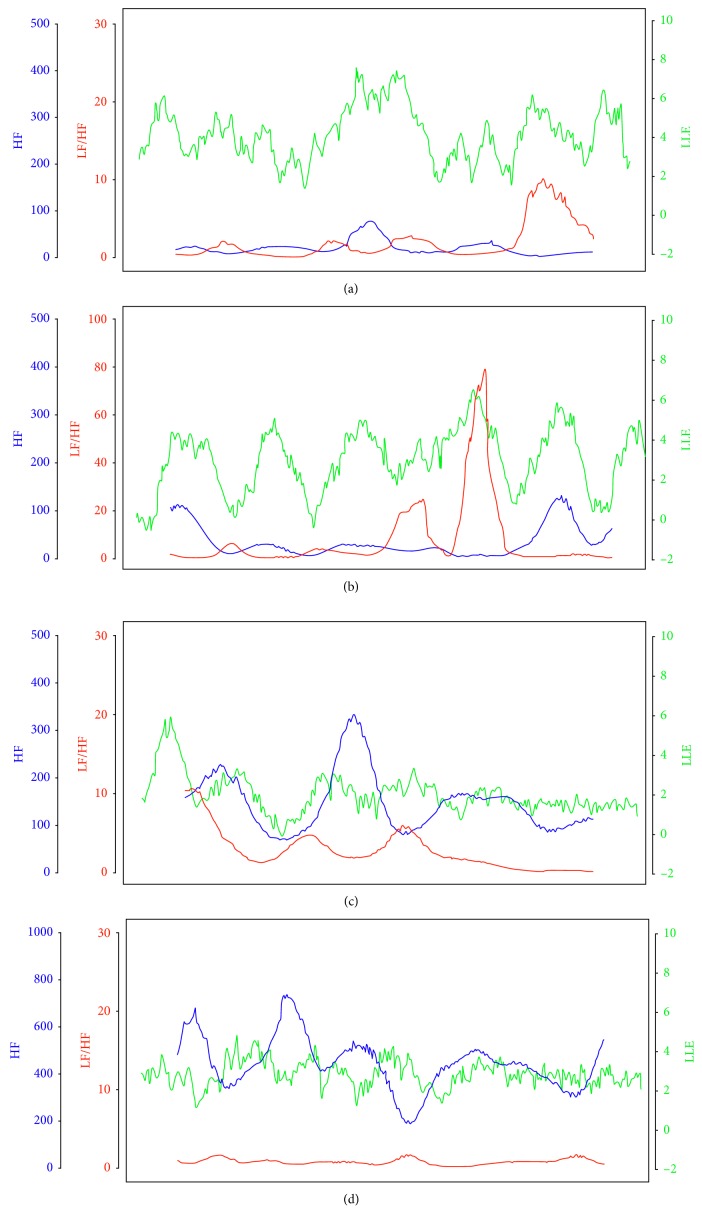
Changes in LLE and HF/LF for pairs of a caretaker and an aged people during communication. (a) Case of Patient A. (b) Case of Patient B. (c) Case of Patient C. (d) Case of Patient D.

From the result shown in Figure 20, we observed that the caretaker's LLE fluctuated normally during the talking, while the LLEs of 4 patients (A, B, C, and D) remained almost unchanged. Looking further for their ANB, we found that the parasympathetic nerve held a dominant position for each of them. As a result, they appeared to be listening but actually were not, and they were possibly asleep even though their eyes were open. Thus, the inability to communicate can also be indicated by the LLE [15].

### 6.10. Comparative Analysis of Mental Disease Sufferers and Healthy People

In order to investigate the possibility of identifying physiological patterns characteristic of mental illness sufferers, we conducted measurement from subjects of mentally ill patients and healthy people [29, 30]. The former group was composed of 43 people, selected from a total of 195 patients each of whom was diagnosed with a certain mental illness. The latter was a group of 133 healthy and young students including 71 females and 42 males, with an average age of 19.6. To enhance credibility, the measurement was carried out several times for each subject.

**Figure 21:**
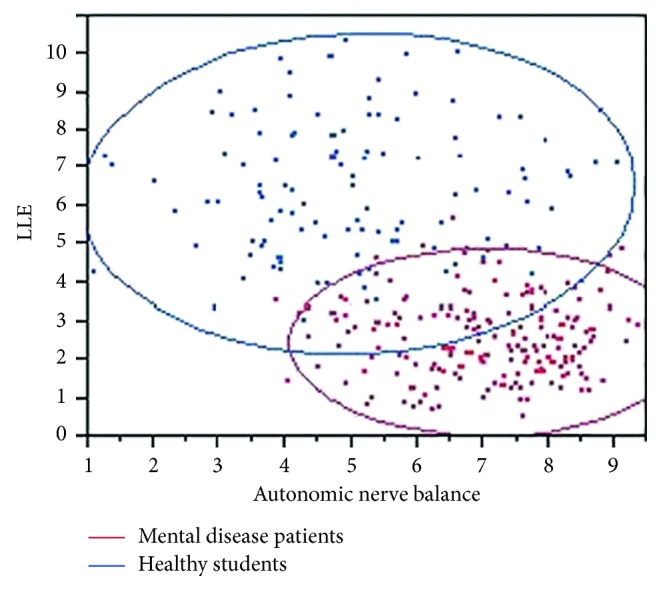
LLE and ANB of healthy (blue) and mentally ill (red) subjects.

After collecting data from the measurement, we observed the analytical results of both LLE and ANB with “Lyspect”, as shown in Figure 21.

**Figure 22:**
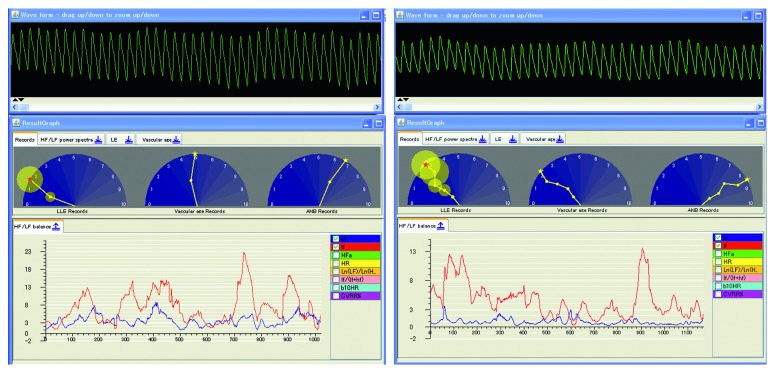
Analytical results of schizoaffective disorder (depressive disorder type) (a) and dysthymic disorder (PTSD) (b).

In particular, we show the results for the subjects suffering dysthymic disorder (PTSD) and schizoaffective disorder (depressive disorder type) in Figure 22. It can be clearly observed that the LLE was always low while the ANB was always higher than 5. This indicated a characteristic of low external adaptability and sympathetic activeness, which is likely to be common to a certain amount of depression-related mental illness suffers.

## 7. Development of Smartphone-Based Device for Self-Check in Real Time

We have developed “Alys,” a mobile application portable for Android tablets and smartphones, which enables users to conduct pulse wave measurement anywhere at any time and display the analytical results in real time during the measurement [6].

This device can even be connected to several sensors for a group of subjects under experiment. In particular, for a simultaneous measurement of a pair of subjects, one subject can observe the real-time result of the other, and vice versa. In this way, we can study the interaction of the two subjects involved in communication.

Furthermore, it can also be used during a meeting to visualize the attendees' degree of concern towards the theme of the meeting. Similarly, for audience rating survey for TV programmes, in addition to the number of audience, some indexes regarding the audience' degree of concern may also be included.

As mentioned above, “Alys” can display the range of LLE values in colour, which enables users to clearly observe the real-time mental status, and then look for ways to keep their LLEs fluctuating within a moderate range. By this means, the users can effectively adapt themselves to the external environment and stay mentally healthy [31].

**Figure 23:**
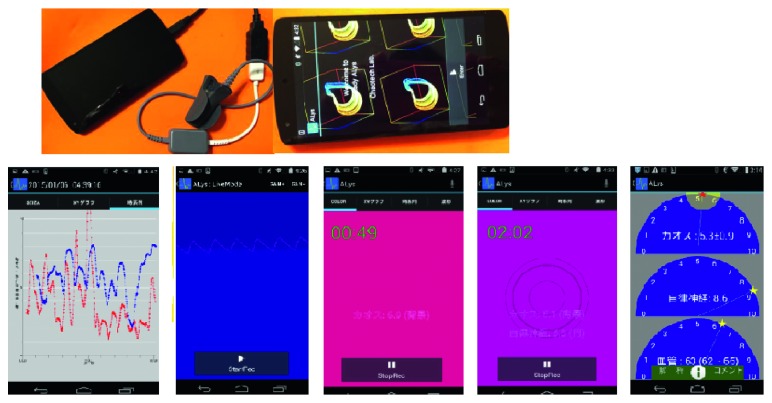
The starting screen and several display screen of “Alys.”

Regarding the output of the analytical results, from display options on the software “CALISMA” which is built in “Alys,” we can show the “mental condition map,” as mentioned in Figure 9 in Section 5.2, which combines the data of both LLE and ANB. Besides, the output can also be displayed as a list of values or semicircular graphs. The output screen of “Alys” is shown in Figure 23.

## 8. Conclusion and Future Prospects

Through the various experiments, we can justify the effectiveness of the largest Lyapunov exponent (LLE) as an indicator of mental immunity. A high LLE indicates a mental status of adapting to the external environment (“external adaptation”). A continuously high level of LLE suggests that the mental immunity of the individual is so strong that he or she is likely to go to extremes: such individuals can be easily irritated and take unexpected actions. On the contrary, a low LLE indicates a mental status of “internal focusing,” and a continuously low level of LLE suggests the mental immunity is so weak that the individual is prone to depression.

Moreover, with the measuring device that we have introduced, measurement with Android tablets or mobile phones can be conducted easily, without the limitation of place and time. The display with intuitive colours makes the values of LLE and ANB more visible. Measurement during various daily activities enables the users to perform real-time mental check-up and then self-control according to his or her mental status.

We are striving for improvement in our future work. Firstly, for mental well-being in such an increasingly complex society, it is beneficial to have a clear grasp of one's mental status, which can be realized through real-time self-check. Thus, sensors that are easy to install and cost-effective are in demand. To this end, we are considering the application of Bluetooth. Moreover, by making good use of the Internet and databases, it is possible to access the past data and then observe the changes within a period of time or with the change of the seasons.

The self-check of mental status can be advantageous to a comfortable living in a complex and stressful society. We cordially hope that this study can contribute to human well-being.

## Figures and Tables

**Table 1:** Number of subjects by age and sex.

	Boy	Girl	Total
0 years old	2	5	7
1 years old	13	10	23
2 years old	19	13	32
3 years old	27	27	54
4 years old	44	25	69
5 years old	34	23	57
Total	139	103	242

**Table 2:** Relationship between day-time LLE and deterioration in willpower, level of anxiety, tendency toward depression, and accumulation of fatigue.

	Willpower decrement	Anxiety	State of depression	Accumulated tiredness	LLE
Willpower decrement	1	0.7235	0.7539	0.7496	**−0.639**
Anxiety	0.7235	1	0.8455	0.9358	**−0.728**
State of depression	0.7539	0.8455	1	0.842	**−0.701**
Accumulated tiredness	0.7496	0.9358	0.842	1	**−0.631**
LLE	**−0.6385**	**−0.7279**	**−0.7014**	**−0.6305**	1

Correlation coefficients are given in bold.
